# The use of a skin-stretching device combined with vacuum sealing drainage for closure of a large skin defect: a case report

**DOI:** 10.1186/s13256-018-1779-8

**Published:** 2018-09-03

**Authors:** Ying Lei, Lei Liu, Si-Heng Du, Zhao-Wen Zong, Lian-Yang Zhang, Qing-Shan Guo

**Affiliations:** Center of Trauma Surgery, Daping Hospital, The Third Military Medical University, State Key Laboratory of Trauma, Burns and Combined Injury, Chongqing, 400042 China

**Keywords:** Skin stretching, Vacuum sealing drainage, Skin defect, Blast injury

## Abstract

**Background:**

This case report presents the treatment of a large infected skin defect, which was caused by an accidental explosion, through a skin-stretching device combined with vacuum sealing drainage. To the best of our knowledge, the area of the wound that we treated may currently be the largest.

**Case presentation:**

A 41-year-old Asian man was transferred to the Center of Trauma Surgery of our hospital for the closure of an open infected wound with a large skin defect in his right lower limb caused by an accidental explosion of 100 pieces of blasting cap. The wounds located in his right gluteal were approximately 40 cm × 35 cm. On admission, the wounds had hemorrhaged, exhibiting a darkened appearance, and included scattered metallic foreign bodies. Debridement of his right gluteal area was conducted 6 hours after injury. Subsequently, a skin-stretching device combined with vacuum sealing drainage was applied to reduce the skin defect. This treatment proved to be valuable for the closure of the skin defect and to attain successful functional rehabilitation without sciatic nerve entrapment or amputation in this case.

**Conclusions:**

It is difficult to close large skin defects, especially when they are infected. The application of a skin-stretching device combined with vacuum sealing drainage should be commonly applied to treat infected wounds because it is a safe and easy operative technique.

## Background

As a widely applied technique for treating wound surfaces, vacuum sealing drainage (VSD) has played an unprecedented role in repairing skin and soft tissue defects, especially in infected wounds of the extremities. However, with a large wound, a split skin graft cannot be avoided, and these grafts not only involve the risks associated with a surgical operation, but also result in the formation of scars in areas of native skin or transplanted skin [1, 2].

Skin-stretching devices (SSDs) were designed to utilize the viscoelastic properties of skin by applying controlled and evenly distributed tension along wound margins by incremental traction. The biomechanical properties of skin, known as mechanical creep and stress relaxation, allow skin to stretch intraoperatively beyond its inherent extensibility in a short period of time. As a result of skin stretching, wound closing tension decreases and allows primary closure of large defects. This technique eliminates problems due to donor defects and associated morbidity. It enables sensate reconstruction with good cosmetic appearance of skin. However, for large infected wounds, the use of a SSD has been limited [3, 4].

Until now, there have been no reports of significantly large wounds that have been directly sutured without additional techniques using gradually reduced attraction material to facilitate skin stretching.

In this case report, a SSD combined with VSD was applied to repair a large skin defect complicated by soft tissues infection in a lower limb, and this technique allowed the patient to finally attain successful functional rehabilitation. This report describes treatment of the largest wound area currently reported in the literature.

## Case presentation

A 41-year-old Asian man was transferred to the Center of Trauma Surgery in our hospital 6 hours after injury for the closure of an open infected wound with a large skin defect in his right lower limb caused by an accidental explosion of 100 pieces of a blasting cap. Hemostasis of the wound was achieved by applying pressure and a total of 2500 ml Ringer's solution, which is a kind of balanced salt solution, was given intravenously during the emergency. He was mildly obese, described himself as quite heathy, and had never been admitted to a hospital previously. He reported no chronic medical history, such as primary hypertension, heart disease, diabetes mellitus, an impaired immune system, malignancies, liver cirrhosis, renal failure, or hemodialysis. He also reported no history of infectious disease, such as tuberculosis, any types of hepatitis, or acquired immunodeficiency syndrome (AIDS). His medical history revealed no trauma, blood transfusion, other surgical procedures, or other serious event. He had not lived in an epidemic area and had no contact history of radioactive exposure. He denied any family history of inherited diseases. He usually did not smoke tobacco or consume alcohol and had no other unhealthy behaviors. He was a business executive and he often traveled for business.

His blood pressure at admission was 99/50 mmHg, pulse rate was 102 beats per minutes, and his respiratory rate was 21 breaths per minute. On examination, his mucous membrane was dry and his conjunctivae were pale. No positive signs were found during neurological, cardiopulmonary, and abdominal examinations. There was no pain around the kidney area with percussion or tenderness along the bilateral ureteral approach.

**Fig. 1:**
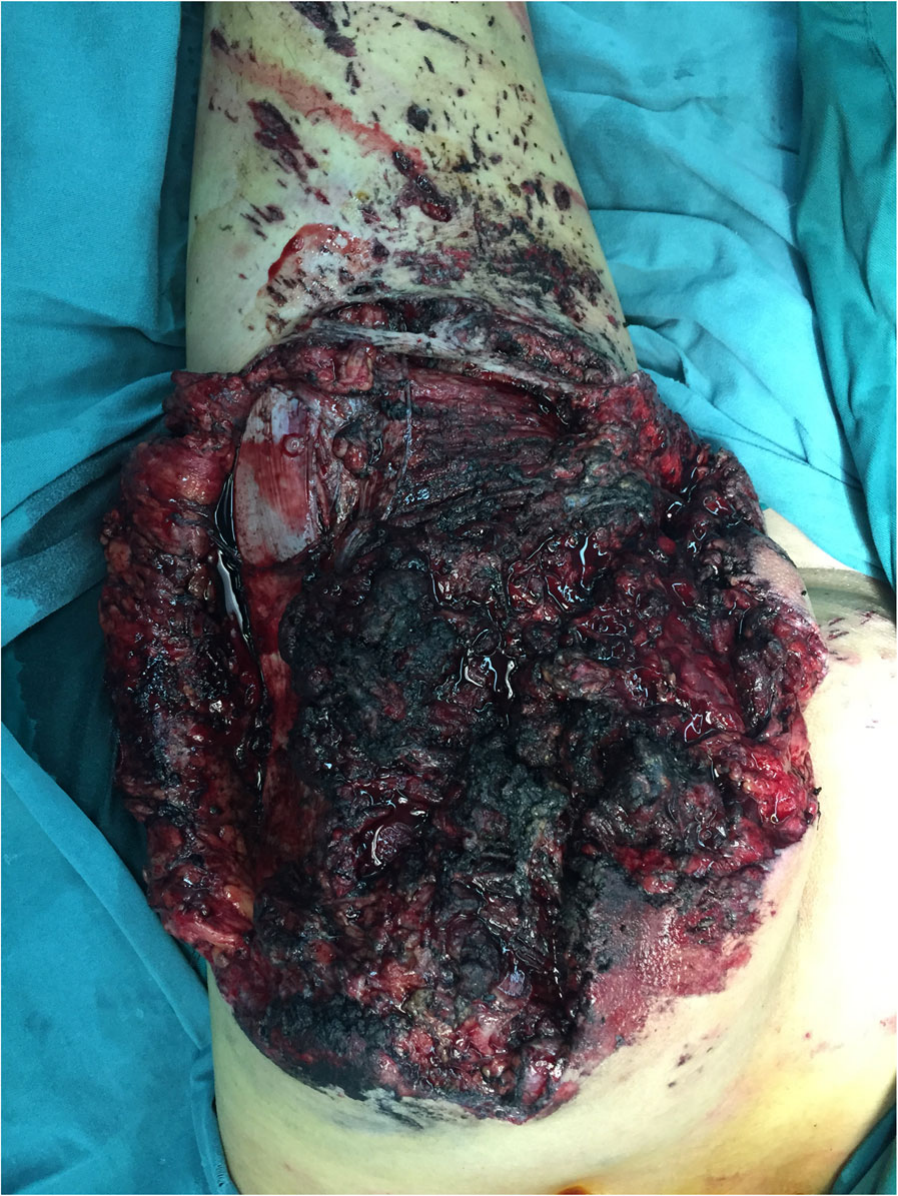
Primary wound of patient at admission

A specialized examination revealed that the wounds were located on his right gluteal and were approximately 40 cm × 35 cm in size with a darkened appearance. The margins of the wounds were 2 cm above the bottom of iliac crest, inferior to the superior segment of back side of his thigh, 3 cm interior of the anal cleft, and external to the lateral thigh (as shown in Fig. 1). The wound had hemorrhaged and contained scattered metallic foreign bodies. Most of his gluteus maximus muscle was injured and the motion of his right hip joint was limited.

In addition, related laboratory examinations were conducted. His complete blood count values were as follows: white blood cell count of 10,940 cells/uL, red blood cell count of 3,250,000 cells/uL, hemoglobin of 9.8 g/dL, and platelet count of 153,000 cells/uL. D-Dimer was 5678μg/L. His total protein was 45.7 g/L, among which the albumin and globulin content were 21 g/L and 24.7 g/L, respectively. The results of serology for renal function were normal. Blood and aerobic and anaerobic bacterial cultures were performed. Microorganisms were not found in the blood cultures. The secretions from injured tissue revealed that a little of the Gram-positive bacteria, *Bacillus subtilis*, was detected. A diagnosis of explosion injury in left gluteal region and hemorrhagic shock was made.

**Fig. 2:**
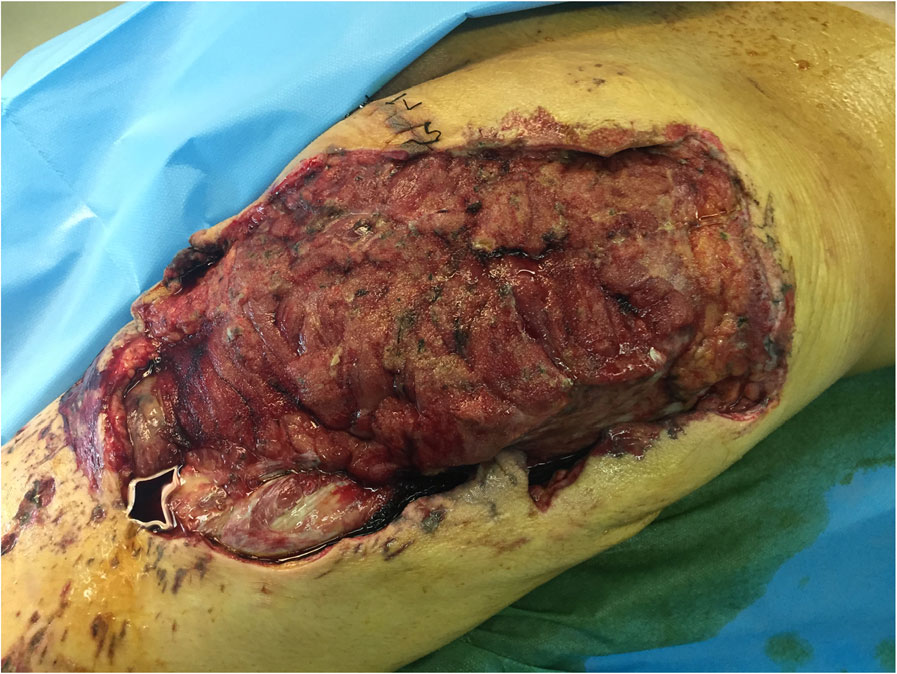
The wound after first debridement

He underwent aggressive fluid administration, hemodynamic support, and intravenously administered antibiotic therapy. Debridement of his right gluteal was carried out 6 hours after the explosion under general anesthesia. Then, the wound was sutured with VSD and adhesive membrane, which finally was connected to negative pressure drainage equipment. During the operation, 800 ml erythrocytes and 400 ml plasma were infused into our patient. Three days after the first operation, he underwent a second operation. The necrotic muscles were excised and then the wound was closed with interrupted suture to shorten the defect to 12 cm × 40 cm (as shown in Fig. 2). The VSD was also connected to the wound as described above.

**Fig. 3:**
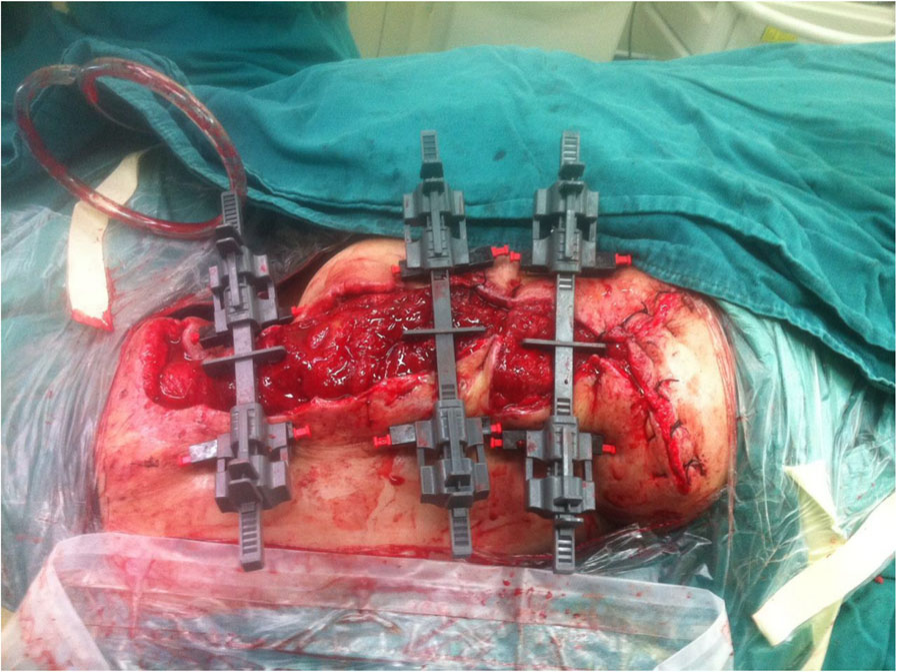
Three skin-stretching devices were applied simultaneously to bring the skin edges together

Nine days after the second treatment, although a few scattered necrotic muscles were located in the wound, the granulation tissues were growing well. The skin around the wound was healthy, with only mild edema and migrated to the wound margins. The pinch test demonstrated that the skin had some mobility, which indicated that it could be sufficiently stretched. Under general anesthesia, the skin margins were minimally free to facilitate the insertion of intradermal needles on both sides of the wound. The wound itself was left undisturbed. Three SSDs (Life Medical Sciences, Inc., Princeton, NJ) were inspected every few hours. The healthy skin was stretched for 4 minutes, followed by 1 minute of relaxation. After stress relaxation had occurred, the tension was adjusted to 3 kg, as indicated by the tension gauge (as shown in Fig. 3).

**Fig. 4:**
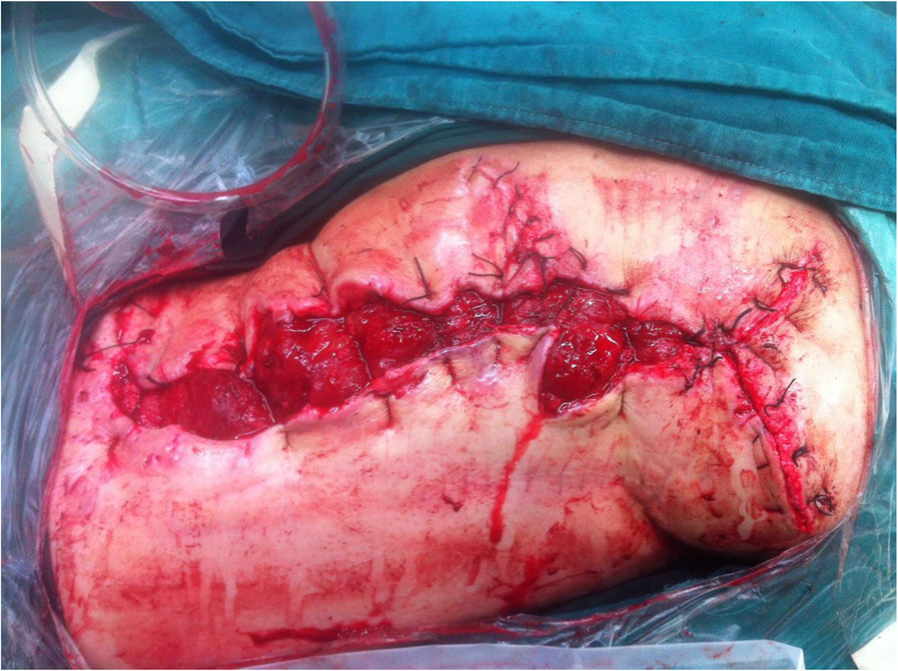
Sutured wound after the first stretching

This procedure was repeated five times during the operation until the skin reached approximation to the wound margins. Then, the devices and the intradermal needles were removed from our patient. During this process, the granulation tissues looked good, the wound was thoroughly irrigated, and the stretched skin margins were closed with interrupted suturing to reduce the size of the defect to 5 cm × 38 cm (as shown in Fig. 4). After stretching treatment, the VSD was applied again to close the wound as before.

**Fig. 5:**
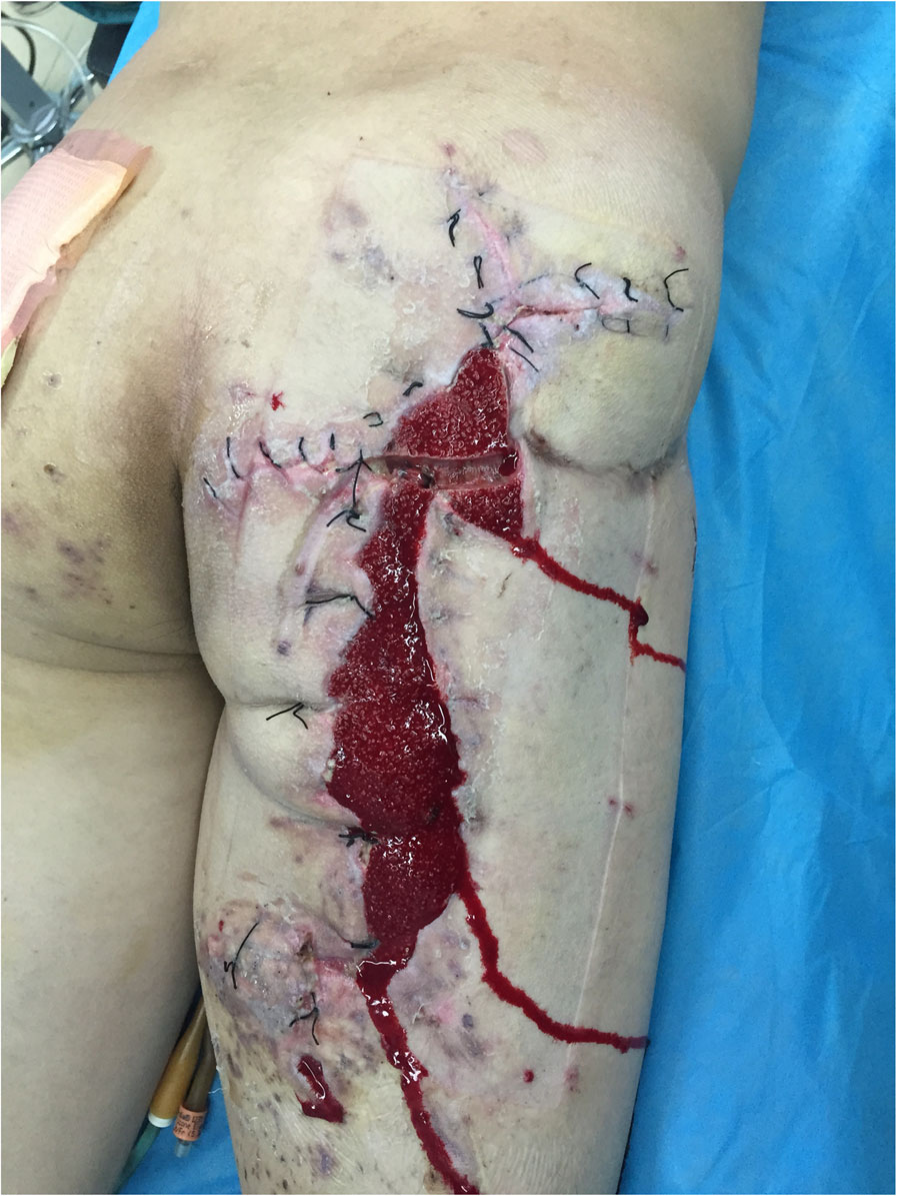
The wound after the second stretching

**Fig. 6:**
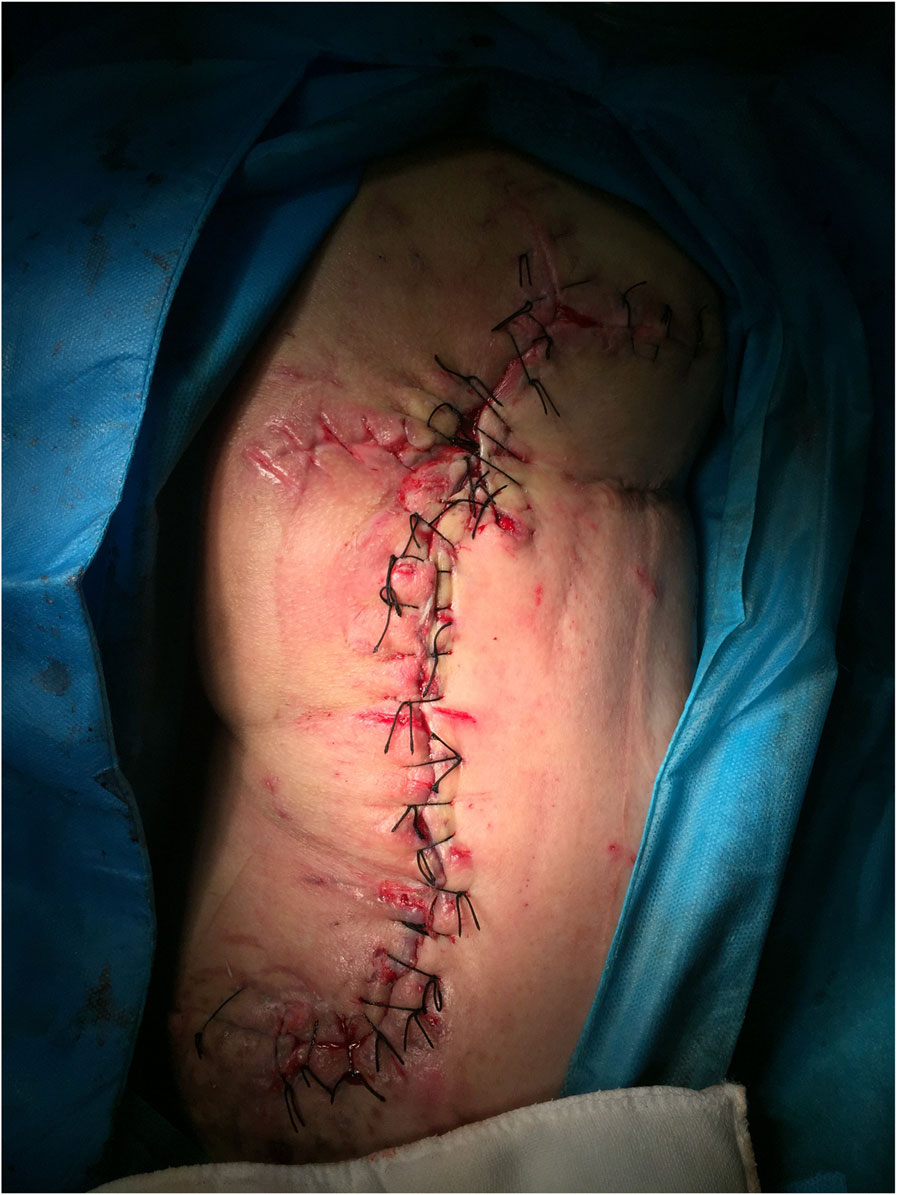
Sutured wound after the second stretching

**Fig. 7:**
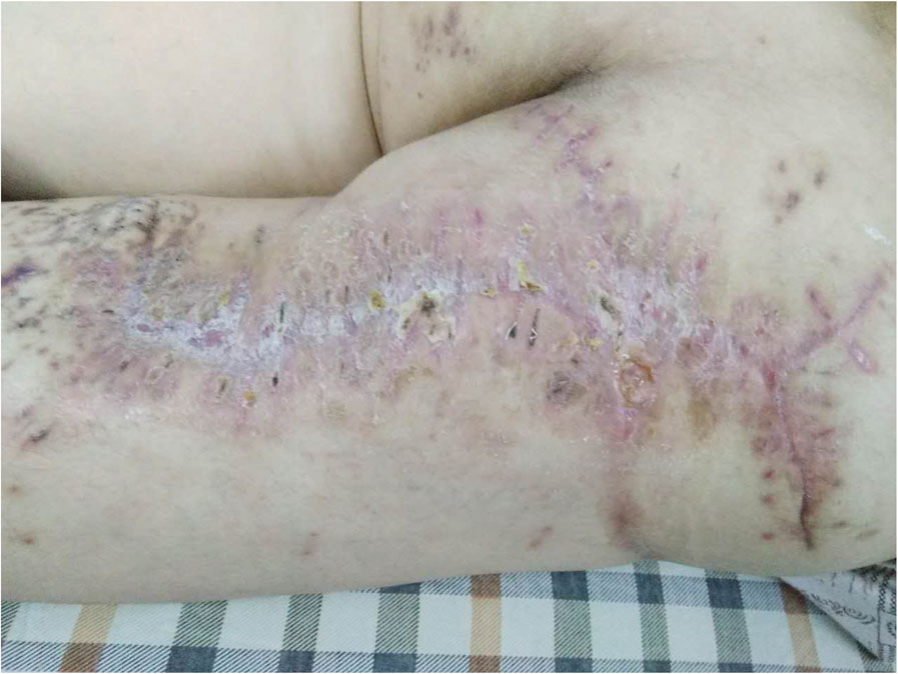
The healing wound of patient at discharge from our hospital

After 9 days, the size of the wound had decreased to 4.5 cm × 35 cm (as shown in Fig. 5). The SSD was then applied again as before. During the last operation, the wound was thoroughly irrigated, and the stretched skin margins were closed with interrupted suturing (as shown in Fig. 6). Eighteen days after this operation, there were only two small wounds that were approximately 1.0 cm × 0.8 cm without edema or inflammation. The local granulation was healthy (as shown in Fig. 7). At that time, our patient was ambulatory. Although he had been in hospital for over 1 month, there was no evidence of damage to the skin margins. The timeline of the patient’s treatment is shown in Table 1.

**Table 1:** Time of the treatment for patient

Time after admission	6 hours	3 days	12 days	21 days	39 days
Treatment	Debridement	Debridement	Skin stretching and VSD	Skin stretching and VSD	The stretched skin margins were interrupted sutured
Result	The wounds were sutured with vacuum sealing drainage and stick membrane	The necrotic muscles were excised and wounds were interrupted sutured	The stretched skin margins were interrupted sutured	Wound was thoroughly irrigated and the stretched skin margins were interrupted sutured	Wound approximately 1.0 cm × 0.8 cm without infection. Granulation tissue is healthy. Patient is ambulatory
Wound Size	40 cm × 35 cm	12 cm × 40 cm	5 cm × 38 cm	4.5 cm × 35 cm	1.0 cm × 0.8 cm

*VSD* vacuum sealing drainage

At 3 months postoperatively the wound was healing perfectly and our patient could walk freely and do some suitable exercise. At 6 months postoperatively, he returned to business work as usual.

## Discussion

This case report presents the treatment of a large infected skin defect, which was caused by an accidental explosion, through a SSD combined with VSD. Debridement of the right gluteal area was conducted 6 hours after injury. This treatment proved to be valuable for the closure of the skin defect and to attain successful functional rehabilitation without sciatic nerve entrapment or amputation.

In recent decades, various SSDs and skin-stretching techniques have become available. Stretching healthy skin can be performed in a preoperative, intraoperative, or postoperative bedside setting. By repeatedly applying intermittent and controlled stretching around the wound edges, healthy skin can be obtained without compromising the blood supply or the quality of the stretched skin. The employment of an SSD assists in supplying full-thickness, healthy, durable, and sensate skin over a weight-bearing area. In addition, this technique avoids the need for skin grafts that are unstable when subjected to weight bearing for tissue expanders and local free flaps [5–7].

In this patient, the skin defect at the wound surface was 40 cm × 35 cm, which was too huge for primary closure. At the same time, the wound was infected, which could have resulted in amputation or one of various types of complicated reconstruction operations without special treatment, so the direct use of a SSD was limited.

Studies have indicated that wounds can be kept relatively clean through local decompression sealing, which can not only decrease the bacteria in wounds and prevent infection, but can also alleviate the edema of granulated tissues and increase blood infusion. In a clinical setting, the usage of VSD can decrease wound surface and control infection, finally promoting wound healing [2, 8]. In this case, after the first debridement, we used only VSD to treat the wound, and interrupted suturing of the wound was delayed for 3 days. Through this treatment, the size of the wound was reduced to 12 cm × 40 cm. Therefore, we repeated the use of a commercially available preoperative SSD to aid the closure of the wound according to the growth of tissues around the wounds. During our serial treatments, it took only 9 days to suture the large wound. It is very difficult to close such large wounds even though the stretching device is used as normal. Through two skin-stretching treatments during our patient’s hospitalization, the wound was reduced from 5 cm × 38 cm to 4.5 cm × 35 cm, and finally only two small wounds of approximately 1.0 cm × 0.8 cm remained. Furthermore, after two operations sciatic nerve entrapment did not occur, and the blood supply to his distal limb was sufficient. The function of his hip joint and knee joint were almost normal through suitable exercise when the wound was closed by debridement.

In this patient, the application of a SSD and VSD shortened his time in hospital, the wound repair had a better appearance, and the additional risk from a skin transplant and the formation of scars was avoided.

## Conclusions

The application of a SSD combined with VSD should be commonly applied to treat wounds that are complicated with infection, as it is a safe and easily reproducible technique, and we believe this technique can be incorporated into the clinical practice of any (plastic) surgeons performing large scar excisions or routinely facing the problem of closing large defects [8, 9].
